# Medical Miscellanies

**Published:** 1817-10

**Authors:** 


### PART IV.

### MEDICAL MISCELLANIES.

### THE PORTE FEUILLE, No. VIII.

OR,

DEPOT FOR DETACHED FACTS AND INTERESTING PHÆNOMENA OCCURRING IN THE PRACTICE OF THE FRIENDS OF THE MEDICO-CHIRURGICAL JOURNAL.

"Ore trahit quodcunque potest atque addit acervo."

*Cauliflower Excrescence of the Penis, with Amputation of that Member.*

Dr. Johnson lately met with a remarkable instance of this disease, which had continued fifteen years, and arose, according to the Gentleman's own account, from venereal warts. The glans penis was transformed into a mass so exquisitely imitating a cauliflower, that no words or drawing could convey half so good an idea of the excrescence, as the vegetable abovementioned. It measured eight inches in circumference, at the widest rim; and its various lobes could be separated to half an inch from the surface. From these indentations, the urine issued in numerous streams, and from these and the whole surface of the excrescence, a most fœtid, semi-purulent discharge continually oozed, with considerable pain and irritation. The prepuce was formed into a thickened, diseased, and indurated ring behind the tumour, and had become, at different times, the seat of abscesses, which left fistulous openings in all directions. Some of these extended along the body of the penis, which was indeed unsound, to within an inch of the pubis. The poor Gentleman's health was greatly undermined by this terrible affection; and lately there had occurred severe hæmorrhages, which threatened to put an end to his existence. Under these circumstances, he put himself under the Reporter's care, who advised the patient to an operation, as the only chance of saving his life, which was now hourly in danger. The glands of the groin were beginning to swell from the constant irritation; and irritative fever was wasting the strength and destroying the appetite and digestion. Emaciation was very considerable. He at length consented to amputation, and it was thus performed the of July 1817. Present Messrs. Creagh and Stewart, Surgeons in the Royal Navy. The tumour being grasped by the latter Gentleman, and the sound part of the penis brought on the stretch, a piece of very narrow tape was passed round it, close as possible to the pubes, and given to Mr. Creagh to draw tight when necessary, and secure the hæmorrhage. Dr. J. now divided the integuments circularly, half an inch before the ligature, and allowed them to contract for a moment. The penis was then divided by a single stroke of the scalpel, moderately close to the integuments. The copora cavernosa and corpus spongiosum instantly shrunk out of sight and reach, and the tape became perfectly useless. The hæmorrhage was considerable. A small sponge was pushed into the hollow, and Dr. J. with the left hand behind the scrotum, pushed forward, as far as possible, the stump of the penis; then withdrawing the sponge, three arteries were seen springing. They were immediately and easily secured, for they had contracted so much less than the surrounding parts, that they projected half a line from the surface. One artery on the dorsum, and one in each corpus spongiosum, were all that bled. Some dry lint was put on the surface, and a T bandage applied. There was no hæmorrhage for an hour; but it then came on, and continued four hours. Every time that the dressings were taken off, and the parts freely exposed, the bleeding from the corpora spongiosa ceased, (there was no artery sending forth blood) but returned when the parts were covered. Pressure with the hand, and some oil of turpentine applied to the wound, restrained the hæmorrhage completely, and the coagulum was allowed to remain till next morning. The patient was then in great uneasiness, having made no water. The dressings were removed, and he immediately passed water freely. The ligatures came away in five days, and in ten days the parts were healed, and the gentleman walking abroad. The orifice of the urethra now appears at the bottom of a hollow cone formed by the integuments, and about half an inch behind the anterior line of the scrotum. No canula or bougie appeared necessary till the end of three weeks, when the orifice closed, and required the introduction of a bougie for some time, till the passage became stationary in calibre.

This disease appears to correspond with the first Case of CANCER PENIS described by Mr. Hey, (chap. xiv.) But it would seem hardly warrantable to apply the name of *cancer* to an ulceration where the parts are continually *increasing in bulk*. Is this the case with cancer in any other part of the body? Mr. Hey remarks, that in seven cases out of nine, there were natural phymoses from birth. Here there was paraphymosis. In the 3d case, where Mr. Hey introduced a canula, after the operation, no good was produced thereby; and Mr. John Pearson's advice to introduce none, seems the best. It also appears quite useless to apply a ligature of tape behind the incisions, since, when the body of the penis is allowed to contract for a few seconds, the arteries will be found projecting so as to be easily taken up, even without hooking them *per tenaculum*.

Although the patient made water perfectly free till the end of the third week, and indeed till the parts were entirely healed, yet, such a firm contraction took place at the orifice of the urethra, that very considerable difficulty was experienced in introducing even the smallest bougie. When once introduced, however, the stricture gave way immediately, and a bougie of any size could be introduced in two days. It therefore seems essentially necessary to keep an eye to the passage as the wound heals, in order to apply the bougie before the contraction becomes so small as to give difficulty in the introduction, as was the case here. J.

---

### *Bleeding in severe Cholera.*

Extract of a Letter from J. B. SHEPPARD, Esq. Surgeon, of Witney, Oxfordshire, to Dr. JOHNSON.

"Your account of Dr. Moulson's Paper brings to my recollection a practice somewhat analogous (though with a different intention) which I pursued during a short service in the Brazils, a few years since, in the violent form of cholera which seems to be endemic there. You have, I believe, described a similar disease in India, under the name of *Mort de Chien*, in which you recommend bleeding, with other remedies; but I have now reference only to the notes which I made of your book, and therefore am not positive. In more than forty cases which came under my care, during the four months we were in the harbour of Rio Janeiro, and on the coast, I found bleeding to *syncope* instantly and uniformly successful *alone*. There was no critical biliary discharge, but the disease was removed before the arm was secured, and no subsequent medicine was required. The intestinal spasm was far more violent than any I had ever witnessed in the West Indies, (where the disease is pretty severe) and bore a strong resemblance to the convulsive paroxysm; so much so, that I was generally called to a man said to be in fits; and the powers of several men were required to restrain them. The first cases I treated by warmth, frictions, volatiles, and opium, but did no good, until I adopted the plan I have mentioned, which, in no instance, disappointed me; the variations of temperature in that climate are extraordinarily great, frequent, and sudden; and to such mutations the prevalence of intestinal spasms may be ascribed."*

---

### *Curious Modification of Variola by previous Vaccine.*

Extract of a Letter from Dr. QUARRIER, of Fort Moncton, to Dr. JOHNSON.

" A fine boy, four years old, son of Serjeant Hutton, R. M. Artillery, was vaccinated three years ago by Mr. Fry, Assistant Surgeon, Royal Marines, Chatham, who has had much experience, and who pronounced it to be a fine specimen of genuine cow-pox. On his arrival at Clay-Hall, in this neighbourhood, the small-pox prevailed, and among others he sickened and received the infection. The febrile excitement did not remain more than two days and he had only about thirty distinct pustules.—He recovered rapidly; but in the mean time, an ACESTORIS in the hamlet inoculated the child of Baker, a gunner, from this boy. I did not see the child till the fifteenth day, when there was a copious eruption of fine pearly small pox, while the areola, where the matter had been insinuated, exhibited every appearance of vaccine inoculation: and had the circumstances been concealed, and the other eruption not shewn, I should have pronounced it to be a beautiful sample of the vaccine areola.

" I shall make no comment on this singular modification after a period of three years had elapsed, but shall leave it to those more versed in physiological science."

---

* Mr. Sheppard will see a striking elucidation of this subject in a case of hydrophobia, by Mr. Webster, related in this Journal. Dr. Sanders, of Edinburgh, has long been investigating these points of pathology, and will, we hope, soon lay the results of his labours before the public. ED.

### MISCELLANIES.

Quicquid agunt Homines.

---

### *Reflections on the Limits between Physic and Surgery.*

By Medico-Tiresias.

Having risen through every gradation of the profession, from an apothecary's assistant to a reputable physician; and having practised each branch of the "art and science" separately, and all combined; I may, like Tiresias of old, be allowed to give an opinion on both sides of the question. I may premise, however, that I did not, as too many do, usurp a higher from a lower grade, without first studying the principles of the branch which I was going to enter on; and I may add, in conclusion, that, however I may have stickled for the honour of the different orders, as I was more or less interested in their dignity, I am now superannuated, and can only look back, like an aged parent on his children and grandchildren, wishing prosperity to all.

The boundaries between Physic and Surgery have not till this moment been defined — a strong presumption that nature does not sanction them! The distinction of internal and external diseases is any thing but correct or satisfactory. Gout, for instance, alternates with erysipelas. In the first form the Physician claims it as his own; in the second, the Surgeon steps in, and demands the treatment. But is not the erysipelas in this case, and indeed in almost every other case, dependent upon an internal, or what is termed a constitutional cause? and can it be, with any degree of safety, treated as a topical affection? Here then, must not Physic and Surgery be united?

The same may be said of anthrax, and a great majority of inflammatory affections, whose prominent and apparent seats are indeed external, but whose cause and origin are internal. If the surgeon treats them without the aid of physic, he does wrong: the physician considers them as out of his province; and if he does undertake the cure, he must use surgical means with medicinal. How preposterous would it be to have a physician combating the cause, and a surgeon the effect — or more correctly speaking, to employ the physician to lop off one arm of the disease, while the surgeon is busy in extirpating the other? Either then the physician and surgeon must be of one opinion, and consequently be equally informed on the subject, or the practitioner must unite physic and surgery in his own person. In short, the distinction between internal and external diseases has no more foundation in nature or truth, than that between diseases of the right and of the left side of the body!

The allotment of all diseases requiring *manual* assistance to the department of surgery would create equal confusion. A patient is seized with violent pneumonia. The physician is sent for: he declares that venæsection is immediately necessary, and the paramount remedial measure. But in these times the surgeon no longer obeys the orders of the physician; they are independent of each other, and act only from their own judgment. But granting that a bleeder is procured to act under the physician, and things go on well for a time, it may and does happen that pneumonia terminates in empyema. Then comes the difficulty. It is surely as necessary (indeed it is much more so!) to know *when*, as how, to perform an operation. The surgeon, on whom the responsibility of the operation rests, must select *his* time for the performance, and for that purpose *ought* to know the nature and exact state of the case as well as the physician. Here physic and surgery must be again united.

On the other hand, let us take the purest surgical case—a compound fracture of the leg. When the surgeon has reduced the fracture, and comfortably adjusted the limb on a pillow, his work is far from being finished. The local will soon produce general derangement, and the whole fabric of the constitution will be in danger without physical aid. Here then, as a contrast to the first example, the physician will be set to work on the *effect*, while the surgeon is occupied with the *cause!* What matchless absurdity! But will it be said, that the febrile commotion excited by the fracture differs in any essential point, either in its nature or treatment, from that occasioned by an internal inflammation, as of the lungs? By no means. Yet the latter is placed exclusively under the physician, and the former under the surgeon. The distinction then, between local and general diseases, cannot be maintained, since organs the most distant are sympathetically influenced, and constantly drawn into consentaneous derangement. All diseases, in fact, are local in their origin. They commence in one organ or set of organs, spreading to all others with more or less rapidity, according to the degree of lesion and importance of function in the organ first affected.

A late eminent French writer (Richerand), who by the bye seems no great friend to the separation of physic from surgery, proposes a new classification of diseases for the more accurately distinguishing the limits of the two branches. All diseases, according to him, may be said to consist in physical derangements, organic affections, and lesions of the vital powers—" lesions des propriétés vitales." To the former only, or *physical derangements*, consisting of wounds, dislocations, obstructions, &c. and their consequent operations, he would confine the province of surgery; leaving a wide field, indeed, to Messieurs les Medecins! " Les lesions physiques sont presque toujours le resultat de l'action d'un corps exterieur sur le notre; ce sont des effets méchaniques d'une cause qui l'est elle-même. Les plaies ou solution de continuité produites par un instrument tranchant, piquant ou contondant; les fractures occasionées par l'alongement forcé du tissu osseux; les luxations amenées par la violence des mouvemens imprimés a nos parties; une hernie, resultat de l'-

effort mechanique des puissances expiratoires, reconnaissent une cause physique, apporte a nos functions un obstacle mechanique, et reclament pour leur guérison, l'emploi des moyens du meme ordre."

According to this scale, the surgeon might still, as he does now, claim the treatment of a chancre, as being the effect of "*un corps exterieur;* but, as soon as the virus had shifted its seat from the prepuce to the tonsil, the physician would say, "Now is my turn!" But does the treatment of a venereal sore in the former situation differ from that in the latter? And if not, why not? seeing that they are treated by different orders of the profession? Here physic and surgery are evidently interlocked. Well may Monsieur Richerand say, "Ces matiéres *Medico-Chirurgicales* peuvent, ce me semble, être comparées à ce crepuscule qui conduit du jour à l'obscurité." But, taking M. Richerand on his own ground, I would ask him, can the external impression of rain, and a piercing cold wind on the surface of the body, checking the perspiration, breaking the balance of the circulation, and inducing pneumonia, be considered strictly, and philosophically, less "*une action d'un corps exterieur sur le notre,*" than the puncture of a bayonet, producing the same effect? Yet, in one case, the inflammation "le *resultat* de l'action, &c." is medical, and in the other it is surgical! What are intermittent fevers—typhus, the plague itself, but the effects of foreign bodies on our own? What is hydrophobia? What is traumatic tetanus, but "*le resultat de l'action, &c.?*"

In no possible way can we find any natural or just distinctions between physic and surgery; and yet I do not object to the distinctions in *practice.* It is against the idea of a difference in *education and study* that the above remarks are levelled. It is morally impossible, that a man can be a good physician without knowing the principles of surgery; and it is still more absurd, to think that a man can be a good surgeon without being intimately acquainted with the principles of physic. If it be objected that an intimate acquaintance with the minutiæ of both branches is too much for one person to acquire, I answer, 1st. in the words of Richerand, "l'etendue de la sciénce ne justifie point les *limites arbitraires* que l'on voulou tracer entre ses diverses parties." 2d. I am of opinion, that it is much more easy to acquire a profound knowledge of both branches, than of either one separately: and for the reasons already stated, that the two sciences are interlocked at all points, even the apparently most distant; for instance, *pneumonia and fracture.* Hence, to attempt to learn the one without the other, is to tie up one of our arms when we commence a mechanical trade!

Is it not absurd to say, that a *moderate* knowledge of anatomy and surgery is useful to the physician, and deny that a *minute* knowledge would be still more so? As for the surgeon, he cannot move a step in safety, without an intimate knowledge of all the laws of the animal œconomy, which the physician studies and observes. His very operations for the removal of diseases that would otherwise prove mortal, are inflictions that often require the most consummate knowledge of physic to counteract. What madness, then, for the surgical student to direct his attention so exclusively, as he too often does, to the study of anatomy and surgery, when he is assured by grey hairs, that nine-tenths of his future professional labours (whatever line he may take) will be what are termed purely medical! Let him compare M. Richerand's list of "Physical Lesions" with the long black catalogue of organic and vital; and he will see that it is but a drop of water in the ocean!

Not that anatomy and surgery are not to be most minutely studied; but I would say, that these branches are only parts of a grand whole, *all* of which must be studied with *equal care*, or every branch is imperfect.

Let us glance at the great disorders of the three principal cavities of the body. Is idiopathic phrenitis more dangerous and difficult to treat than that from a fracture of the skull? Are idiopathic convulsions more terrible than those from a splinter of bone driven through the coverings of the encephalon? Is idiopathic tetanus more fatal than traumatic? In what consists the difference between idiopathic and traumatic pleuritis, pulmonitis, carditis, diaphramitis, hepatitis, gastritis, splenitis, enteritis, peritonitis, cystitis?—None. Or, if there be any difference, the difficulty and the danger are on the side of surgery!

What then remains? Fever? Does the hectic of the lungs exhibit a greater spectacle of horror and emaciation than the hectic of lumbar abscess, or disease of the hip-joint? Is the inflammatory fever resulting from aërial vicissitudes to be compared with that supervening on a lacerated member or crashed knee-joint, where the dire commotion threatens every instant to annihilate the living fabric at a blow? The typhoid fevers differ in no respect from the ulterior stages of inflammatory and symptomatic fevers.

Seeing, then, that the greatest and most dangerous diseases, both in physic and surgery, to which humanity is subject, are completely identified, is it not evident, that the surgeon, in acquiring a knowledge of *these*, must, in course, acquire a knowledge of the minor and intermediate maladies, which are reckoned *Medico-Chirurgical* by both professions? And, is it not evident from these premises, that the surgeon, if properly qualified, must add to the *science* of the physician the *art* of operating? Anatomy, physiology, and surgery, are merely the first steps by which the student gains a footing on the hill of science. When he can unravel the brain without a light, or take up the external iliac blindfold, he is scarcely advanced a day's march on the great journey of medical science! These, what are termed, and justly, elementary branches, are little more than the alphabet of medicine, though they are considered at the beginning as the ultima thule of professional knowledge. The surgical student thinks himself armed at all points; but every year's subsequent experience shews him more and more his own ignorance!

In fine, physic and surgery are only parts of a grand whole; one cannot be properly known without the other, no more than navigation can be learnt without the aid of arithmetic. The distinctions between the two branches cannot be drawn or maintained, even in the largest cities. To be convinced of this, let any one sit down by the side of Cooper and Abernethy for a day, and he will see these illustrious surgeons lopping off nine surgical diseases with the quicksilver pill and infusion of cascarilla, for every one which they remove by the knife or by caustic. But what is more extraordinary, he will see patients of all ranks afflicted with purely *physical* diseases, resort to surgeons for advice. Hence, it is evident, that the general sense of mankind acknowledges not the artificial divisions which we have made in the profession.

Nevertheless, the separation of physic from *operative* surgery, whatever violence it may do to Nature, is probably productive of good to both branches. There are few minds capable of resisting the seductions of the scalpel, where a bold and surgical genius is bestowed by nature. The eclat of operations eclipses the slow, and dull, and too often unsatisfactory march of medical science; and constant application to one line begets, of course, a facility and dexterity that cannot be otherwise attained. The deficit in other branches is not felt in a great city or public institution, where the patient is surrounded by all ranks and degrees of the Faculty. On the other hand, the physician, liberated from the anxieties of the operation room, brings a calm unruffled mind to the investigation of the infinitely varied phænomena of disease. A close attention to these generates, without a doubt, a quickness of apprehension, a fertility of resource, and an accuracy of diagnosis, to which but the *favoured few* of surgeons can pretend. But, except the purely *operative* part of surgery, medical and surgical *practice* is completely blended, for the best of all reasons, that medical and surgical diseases are essentially the same. Hence, it is incumbent on the young physician to *study* surgery at least, and to dissect as assiduously as though he were designed for the operation room; while the surgical student may be assured that *medicine* is his sheet anchor at last; and that whatever degree of accuracy he may attain as an anatomist, or dexterity as a surgeon, yet, without pathology and therapeutics, these brilliant acquisitions will avail him little in his professional journey through life.

MEDICO-TIRESIAS.

---

### *To the Editors of the Medico-Chirurgical Journal & Review.*

GENTLEMEN,

WHEN I read Dr. Johnson's remarks on the *dictum* so authoritatively advanced by Dr. Balfour respecting Gout (*Medico-Chir. Journal for May*, 1817. *Art.* MEDICAL OBSERVER) I could not but join in the justness of the reprehension it conveyed. Judge then of my surprize, in finding in your number for July, a retraction of that just censure, upon the ground, that the term

"conceit," used by Dr. Balfour, was not, as at first thought, directed against the MEDICAL PROFESSION, but *(risum teneatis)* against the popular opinion, sanctioned by the first medical authorities!" Now, I profess that my intellects are too dull to perceive, how this *new reading* could at all lessen the charge of ignorance and untruth, which Dr. Johnson at first believed, and justly believed, to have been levelled against the profession at large by Dr. Balfour; for, if the popular opinion be (as Dr. B. asserts) "a conceit, and deserving no better name" (see his letter), assuredly the faculty in sanctioning it, are deserving of every blame which such an opinion draws with it; inasmuch as *they* are the only fit and proper judges of its truth or falsity. I cannot help believing, then, that Dr. Johnson, upon re-considering the matter, will see reason to retract his *hasty concession* of having bestowed a *hasty censure* upon Dr. B.

As to the question itself, whether gouty patients experience an improvement both in bodily and mental power after a regular attack upon the lower extremities, sanctioned, as we affirm it to have been, by the concurring testimony of the best authorities, viz. by persons actually labouring under the disease, both professional men and others,—Dr. Balfour must display greater proofs of originality than we have yet witnessed in his lucubrations, to make us concede any greater claim, than that of "conceit," either to him, or to any other of those new luminaries in medicine, who, like him, would have us believe, that gout need not be borne, because it admits of being *cured* like fever, or any other casual complaint. With equal truth and justice, there are some who unblushingly persist in holding forth to the public, that *they*, aided by the wonder-working balsamic atmosphere of a German stove, can cure *phthishis pulmonalis;* converting into wood, that which by immersion in a petrifying spring had become stone; in other words, they would persuade the unfortunate and credulous patients and their friends, who, like drowning men, catch at a straw, that they can restore tuberculated lungs to their natural structure and function!

But, (to return to our subject) without considering it worth while to notice Dr. Balfour's claims to originality, in the pathothological principle and practice of uniting severed parts,—which had been so long ludicrously immortalized by Butler in his Hudibras, as due to Taliacotius, he has also claimed the merit of being the first to cure gout and rheumatism by the *remedium baculinum,* or, in plain English, by squeezing and beating the inflamed parts. Perhaps there would accrue neither gain nor loss to the world in general, and to the medical world in particular, by allowing Dr. Balfour to wear this cap and bells, without disputing with him the honour of priority. But I am an old-fashioned man, who holds the principle of "*cuique suum,*" *i. e.* "Give the devil his due!" to be strict justice, and of course applicable to the most insignificant, as well as to the greatest claims.

On this ground I am compelled to say, that there exists a *medical* competitor, in the person of a Mr. Hayton, a practitioner of pharmacy and midwifery, who formerly resided, and perhapsstill resides, in Charlotte Street, Blackfriar's Road.* This gentleman (himself a gouty subject, and in so far a better testimony of the alleged efficacy of mechanical percussion in gout) printed and distributed, about nine or ten years ago, a small paper, of which I received a copy ; assuring the faculty, that he had frequently removed gout from his foot, by repeatedly striking the *sole* (not the inflamed part) with a thin, flat, and broad ruler,. somewhat (I conceive) like a Harlequin's wooden sword, and, according to his account, endowed with all the magical effects of that instrument, so often presented to our wondering eyes during the Christmas holidays, viz. of making the blind to see, the lame to walk, and even the dead to rise "with twenty mortal murthers on their crowns." So far then, and with good reason, is Dr. Balfour deprived of all claim to *priority ;* nor are his pretensions greater as to *probability* of success ; for we can admit, that a smart blow upon the *sole*, with such a light instrument, if not *beneficial*, could at least be more easily borne, than squeezing or striking the inflamed part.—But, alas ! we are born to disappointment, and find every day the truth of Solomon's remark, that—"there is nothing new under the sun ;" for in the course of my reading for amusement, I have found, that the rightful claimant to this wonderful discovery, even before Mr. Hayton, was a Turkish practitioner ; who, no doubt, derived his medical degree at that infallible Mussulman school, Caaba at Mecca. Baron de Tott, in his entertaining Memoirs, tells us, that a Christian merchant being severely attacked with gout, a Bassa, who was his friend, sent a message to assure him of a speedy cure, which the messenger was commanded to administer : this was, neither more nor less than *fifty* bastinadoes on the soles of the feet ! The merchant, who did not at all relish the remedy proposed, returned grateful thanks to the Bassa for his kind attention, but assured him, that he was already much better, and hoped soon to be well without the profered assistance. But the Bashaw was not to be so satisfied, and insisted on the administration of his remedy ; with which the merchant was obliged to comply, and had the satisfaction to find, that in a few days after, he was able to visit his Turkish highness *on foot*, and thank him for the speedy and complete relief which he had experienced from his prescription.

Old Q in the Corner.

*August 26th*, 1817.

Dr. Johnson begs to say, that he has not retracted his opinions on the *pathological* points discussed ; but he was anxious to with-

---

* N. B. Not Dr. Haighton of St. Saviour's Church Yard, Southwark ; the able Professor of Physiology and Midwifery in the Medical Shcool of Guy's Hospital.

draw any expression that could hurt the feelings of a fellow practitioner, or be construed into personal enmity.

---

*New Method of detecting Arsenious Acid, or Corrosive Sublimate, when in Solution.*

"Take a little recent wheat starch; add to it a sufficient quantity of iodine to give it a blue colour; mix a little of this blue matter with water, so as to have a blue-coloured liquid. If into this liquid a few drops of an aqueous soloution of arsenious acid be poured, the blue colour is immediately changed to a reddish-brown, and is gradually dissipated entirely.

"The solution of corrosive sublimate produces nearly the same effect; but if some drops of sulphuric acid be added, the blue colour is again restored, if it has been destroyed by arsenious acid; but if it has been destroyed by corrosive sublimate, it is not restored either by sulphuric acid, or by any other acid."—*(Brugnatelli Annal. de Chimie et Phys.)* iv. 334.

We have not had an opportunity of trying the above test; but in the mean time present it to our readers on account of the respectable source from whence we have taken it. We are firmly of opinion, that liquid tests of arsenic are merely corroborative—not conclusive: and that no conscientious medical man could give evidence (that might affect the life of a fellow-creature,) deduced from these tests, or from any thing, indeed, short of reduction of the metal.—Editors.

---

Mr. Gaulter will deliver, in the ensuing season, Two Courses of Lectures on the Physiology of the Human Body, at No. 10, Frith Street, Soho Square. The Lectures will be given on Monday and Thursday evenings at a quarter past eight o'clock, after the Surgical Lectures are concluded. The Introductory Lecture of the First Course, will be on Thursday, the 9th of October.

Mr. Guthrie, Deputy Inspector of Military Hospitals, will commence his Autumnal Course of Lectures on Surgery, on Monday the 6th of October, at five minutes past eight in the evening, in the Waiting Room of the Royal Westminster Infirmary for Diseases of the Eye, Mary-le-bone Street, Piccadilly. To be continued on Mondays, Wednesdays, and Fridays.—Medical Officers of the Navy, the Army, and the Ordnance, will be admitted gratis, on obtaining a recommendation from the Heads of their respective departments, which must be presented to Mr. Guthrie between the hours of two and half past four, at his house, No. 2, Berkeley Street, Berkeley Square.

Mr. T. J. Pettigrew, F. L. S. Surgeon Extraordinary to their Royal Highnesses the Dukes of Kent and Sussex, will commence his Winter Course of Lectures on Anatomy, Physiology, and Pathology on Friday, the 17th of October, at eight o'clock in the evening, precisely. The Lectures will be continued every succeeding Wednesday and Friday, at the same hour, until completed.—Particulars may be known, by applying to Mr. P. Bolt Court, Fleet Street.

Dr. Ramsbotham will begin his Lectures on Midwifery, and the subjects connected therewith, on Monday, October 6, at eleven o'clock in the morning, in the Theatre of the London Hospital. Particulars may be had at his house, 37, Broad Street Buildings; and of Mr. Jenkinson, Apothecary to the Hospital.

Dr. Uwins, Physician to the City and Caledonian Dispensaries, will read an Introductory Lecture to a Course on the Theory and Practice of Medicine, at his house, No. 1, Thavies Inn, on Friday evening, the 3d of October, at seven o'clock precisely; and will continue the Lectures every Monday, Wednesday, and Friday evenings, until the conclusion of the Course.

Mr. Thomas Bell, of Fenchurch Street, Surgeon-Dentist, has been appointed Lecturer on the Structure and Diseases of the Teeth, at Guy's Hospital, in the stead of the late Mr. Fox.

On Thursday, the 28th of last month, Alexander Copeland Hutchison, Esq. late principal Surgeon to the Naval Hospital at Deal, was elected, after a severe contest, one of the Surgeons to the Westminster General Dispensary, vacant by the resignation of Thomas Copeland, Esq.—The other candidates were, Mr. George Babington, Surgeon to the Lock Hospital; and Mr. Benjamin Golding.

Died.—At his house in Serjeants' Inn, Fleet Street, William Charles Wells, M. D. F. R. S. L. and E. and one of the Physicians to St. Thomas's Hospital.—In Hanover Square, aged 73, Sir James Earle, Knt. Surgeon to his Majesty, Master of the Royal College of Surgeons, and Senior Surgeon to St. Bartholomew's Hospital.—Mr. Berry, Surgeon, of Falkingham.

